# Time to Death and Associated Factors among Tuberculosis Patients in South West Ethiopia: Application of Shared Frailty Model

**DOI:** 10.3390/diseases10030051

**Published:** 2022-08-05

**Authors:** Yasin Negash Jabir, Tafere Tilahun Aniley, Reta Habtamu Bacha, Legesse Kassa Debusho, Teshita Uke Chikako, John Elvis Hagan, Abdul-Aziz Seidu, Bright Opoku Ahinkorah

**Affiliations:** 1 Department of Statistics, College of Natural Sciences, Jimma University, Jimma P.O. Box 378, Ethiopia; 2 Department of Statistics, University of South Africa (UNISA), Christiaan de Wet Road & Pioneer Avenue, Johannesburg 1709, South Africa; 3 Wondo Genet College of Forestry and Natural Resource, Hawassa University, Hawassa P.O. Box 05, Ethiopia; 4 Department of Health, Physical Education and Recreation, University of Cape Coast, Cape Coast PMB TF0494, Ghana; 5 Neurocognition and Action-Biomechanics-Research Group, Faculty of Psychology and Sport Sciences, Bielefeld University, Postfach 10 01 31, 33501 Bielefeld, Germany; 6 Centre for Gender and Advocacy, Takoradi Technical University, Takoradi P.O. Box 256, Ghana; 7 College of Public Health, Medical and Veterinary Sciences, James Cook University, Townsville, QLD 4811, Australia; 8 School of Public Health, Faculty of Health, University of Technology Sydney, Sydney, NSW 2007, Australia

**Keywords:** parametric shared frailty model, survival analysis, time to death, tuberculosis

## Abstract

(1) Background: Tuberculosis is a bacterial disease mainly caused by *Mycobacterium tuberculosis*. It is one of the major public health problems in the world and now ranks alongside human immunodeficiency virus (HIV) as the leading infectious cause of death. The objective of this study was to investigate the potential risk factors affecting the time to death of TB patients in southwest Ethiopia using parametric shared frailty models. (2) Methods: A retrospective study design was used to collect monthly records of TB patients in three selected hospitals in southwest Ethiopia. The data used in the study were obtained from patients who took part in the directly observed treatment, short-course (DOTS) program from 1 January 2015 to 31 December 2019. The survival probability was analyzed by the Kaplan–Meier method. Log-rank tests and parametric shared frailty models were applied to investigate factors associated with death during TB treatment. (3) Results: Out of the total sample of 604 registered TB patients, 46 (7.6%) died during the study period and 558 (92.4%) were censored. It was found that the median time of death for TB patients was 5 months. Hospitals were used to assess the cluster effect of the frailty model. A Gamma shared frailty model with Weibull distribution for baseline hazard function was selected among all models considered and was used for this study. It was found that the covariates, age, initial weight, extrapulmonary type of TB patient, patient category, and HIV status of TB patient were significant risk factors associated with death status among TB patients. (4) Conclusions: The risk of death was high, especially with cases of HIV co-infected, retreated, and returned-after-treatment categories of TB patients. During the treatment period, the risk of death was high for older TB patients and patients with low baseline body weight measurements. Therefore, health professionals should focus on the identified factors to improve the survival time of TB patients.

## 1. Introduction

Tuberculosis (TB) is a bacterial disease mainly caused by *Mycobacterium tuberculosis* [1]. It remains one of the major public health problems and now ranks, together with human immunodeficiency virus (HIV), as the leading infectious cause of death [2]. Globally, there were an estimated 10 million people who developed active TB disease in 2019 [3], with 1.5 million people estimated to have died from TB worldwide in the same year, whereas 26.7% million were HIV-positive. The global tuberculosis report in 2018 showed that the incidence rate in developed countries was less than 0.01%. The TB incidence rate is decreasing, but not sufficiently rapidly to achieve the 2020 milestone of a 20% reduction in incidence between 2015 and 2020. A nine percent reduction was observed from 2015 to 2019, including a reduction of 2.3% between 2018 and 2019 [4], with incidence ranging from 0.15% to 0.4% in most high-TB-burden countries [5].

In developed countries with good national TB control programs, no more than 10% of cases die during the follow-up period, while in high-resource settings, most cases of deaths that occur during TB treatment are often because of causes other than TB [6]. In sub-Saharan African countries, about 70% of patients with smear-positive pulmonary TB are HIV-positive and the death rate has been increasing for the last 10 to 15 years [4]. A study undertaken in rural Malawi showed that about 77% of TB patients were HIV-positive [7]. Around 20% to 30% of HIV-infected patients died due to smear-positive pulmonary tuberculosis within 12 months of starting treatment [8].

Even though Ethiopia has applied the directly observed treatment, short-course (DOTS) plan since 1992, TB in Ethiopia is considered to be a major public health problem. At present, the preventative approach to TB in Ethiopia depends on the WHO-recommended “Stop TB Strategy” which has been applied in the country since 2006 [9]. The 2018 WHO TB report [5] showed that Ethiopia ranked as the 7th most highly TB-affected country in Africa and was represented in the list of the world’s 22 high burden countries, with an estimated TB incidence rate of 379 for all forms of TB and 168 for smear-positive TB per 100,000 people. Based on the national population TB prevalence survey in 2010 to 2011, it was found that the prevalence of bacteriologically confirmed cases was 156 per 100,000 population, and the prevalence of all forms of TB cases was 240 per 100,000 population [10].

As in many other resource-limited countries, treatment outcomes for tuberculosis have not been satisfactory, mainly due to poor treatment compliance and low coverage of short-course chemotherapy [11]. Comorbidity, massive tuberculosis, and respiratory failure haemoptysis are the main causes of death in tuberculosis patients [12]. Many efforts have been made by the Ethiopian Ministry of Health and its stakeholders to reduce health problems. Among these efforts, the Health Management Information System (HMIS) established a macro-plan to improve staff performance in TB service delivery [13]. However, the epidemiological factors influencing tuberculosis in Ethiopia have not been well studied. It is believed that, in resource-poor countries such as Ethiopia, the survival of TB patients under the DOTS program depends on a variety of factors, which vary greatly with socio-demographic, behavioural risk and health factors [14,15,16].

According to the literature, risk factors associated with reduced survival time of TB patients under the DOTS program are age, initial weight, TB category of patient, type of TB and HIV status, e.g., see [6,15,17]. Further risk factors associated with the survival time of TB patients include the gender of the patient, and whether the tuberculosis is classified as positive-pulmonary, negative-pulmonary or extrapulmonary (e.g., see [18,19,20]). The Ethiopian Ministry of Health [13] promotes the “Stop TB strategy” in all health centers in the form of counselling and regular follow-up of TB patients. When health professionals diagnose a patient with common symptoms of tuberculosis, the patient is referred to the nearest available hospital for further examination.

The objective of this study was to identify the risk factors that affect the time to death of TB patients using parametric shared frailty models. Data for TB patients considered in this study were collected regularly until the patient was fully recovered, was lost to follow-up or died due to the effect of TB.

## 2. Methods

### 2.1. Data Source, Sampling Design, and Sample Size

This was a retrospective study conducted in the TB and HIV outpatient clinics of three randomly selected southwest Ethiopian public hospitals. In total, there are seven teaching and practical hospitals which provide services to the south-western part of the Oromia region, some parts of the Southern Nations and Nationalities, and the Gambella regions of Ethiopia. The three hospitals, Bedelle, Mizan Aman, and Bonga were selected using a simple random sampling technique [21]. The study population consisted of all TB infected patients visiting the selected hospital HIV and TB outpatient clinics for their regular follow up and who were being treated between January 2015 and 31 December 2019. The duration of treatment for patients varied from 6 to 8 months. Treatment outcomes were measured for all registered patients within a two month period after the start of treatment.

Applying techniques described by Cochran [21], a total sample size of 641 was determined assuming a 95% confidence level, a 15% prevalence rate of TB [16], and 0.0281 margin of error. Then, equal numbers of patients were randomly selected from each selected hospital and data were collected from the patients’ follow up records. There were 37 patients with incomplete information on their epidemiological, clinical and laboratory results who were excluded from the study. Therefore, a total of 604 patients were considered to be eligible for the study. To abstract information from patients’ follow up records, nurses were recruited and were trained by the investigators. In addition, the process of data collection was regularly supervised by the investigators to ensure the completeness, consistency, and accuracy of the data.

### 2.2. Institutional Review Board Statement

Ethical approval and human subject research approval for the study was received from Jimma University, College of Natural Sciences Research and Ethics Review Board (RERB), and the medical director of the hospital. As the study was retrospective, the requirement for written informed consent of study participants was waived by the Research and Ethics Review Board (RERB) of the College of Natural Sciences, Jimma University, but data obtained were kept anonymous and confidential.

### 2.3. Inclusion and Exclusion Criteria of the Patients

All patients who were treated for TB according to the national TB treatment guidelines from January 2015 to 31 December 2019, and were registered in the TB registry log book with known treatment outcomes, were included in the study. Patients who did not have a full history of their epidemiological, clinical, and laboratory results were excluded from the study.

### 2.4. Study Variables

The response variable considered in this study was the time to death or the survival time of TB patients with an indicator outcome coded as 1 for “death” or 0 for “censored” events. The survival time was calculated as months from the date of start of TB treatment to the date of the last visit to the treatment center or death. To investigate the effect of prognostic and risk factors on the overall survival of TB patients, covariates known to affect survival in relation to tuberculosis were measured. These covariates were classified as socio-demographic and clinical covariates. The demographic covariates were: sex, categorized as male or female; the residence of patients, categorized as urban or rural; baseline age in years; and the initial weight of the TB patient. The clinical covariates were: type of tuberculosis, categorized as smear-negative, smear-positive or extra-pulmonary tuberculosis; category of patient, categorized as new, re-treated or return after default; history of previous treatment, classified as yes or no; HIV status, classified as positive or negative; and sputum results, classified as a negative or positive result.

### 2.5. Statistical Analysis

Survival analysis refers to the statistical data analysis of the time to an event or survival outcome. Survival data are almost always incomplete in some way or are censored. The censoring may be right, left or interval censoring. The most common type of censoring is right censoring, which applies when the event occurs after the observed survival time or follow-up time [22]. Survival data can be represented using life tables [22], Kaplan–Meier curves [23] and median survival times  [24]. Apart from the estimation of survival functions, comparison of two or more estimated survival curves has been the most commonly used statistical tool in recent clinical research [23].

In the current study, the Kaplan–Meier method and the log-rank test were used to compare the survival times between two or more groups. Under the null hypothesis that there are no differences in the survival functions between *k* groups of survival data, the log-rank and Breslow test statistics have a $\chi^{2}\left(k-1\right)$ distribution [23]. However, to explore the relationship between individual survival and the explanatory variables, an approach based on statistical modeling was used.

Thus, to model and analyse the time to death of TB patients, we considered various parametric shared frailty models. Frailties are random effects accounting for possible association or unobserved heterogeneity of clustered survival data. In a shared frailty model, the lifetimes of a group of observations in the same cluster share the same level of frailty, that is, the common frailty variance measures of dependence among lifetimes within a cluster  [25,26].

#### 2.5.1. Shared Frailty Model

Suppose that there are *m* clusters and each cluster *i* has $n_{i}$ observations with a total sample size $\sum_{i=1}^{m}n_{i}=n$. Let $t_{ij}=min\left(c_{ij},t_{ij}^{*}\right)$ be the observed failure time with a right censoring scheme for the $j^{th}\left(j=1,2,\ldots ,n_{i}\right)$ observation in the $i^{th}$ cluster, and $c_{ij}$ be the censoring time; it is assumed that the failure time $t_{ij}^{*}$ and censoring time $c_{ij}$ are independently distributed random variables [25]. In addition, let the random variable $\nu_{i}$ be the frailty effect of the ith cluster with known distribution function $f_{\nu }\left(\nu_{i}\right)$. Conditional on the frailty variable $\nu_{i}$ and covariates $x_{ij}$, the survival function $S_{ij}\left(.|x_{ij},\nu_{i}\right)$ at time *t* of the jth observation of cluster *i*, is given by [27];

(1) $$S_{ij}\left(t|x_{ij},\nu_{i}\right)=exp\left\{-\Lambda_{0}\left(t\right)\nu_{i}e^{x_{ij}^{\prime }\beta }\right\},$$

where, $\Lambda_{0}\left(t\right)$ is the cumulative baseline hazard function which needs to be specified for generating survival data, and β represents the unknown regression parameters. For invertible $\Lambda_{0}\left(t\right)$, the failure times can be computed by:

$$t_{ij}^{*}=\Lambda_{0}^{-1}\left\{-ln\left(U_{ij}\right)e^{-\frac{x_{ij}^{\prime }\beta }{\nu_{i}}}\right\},$$

where, $U_{ij}∼U\left(0,1\right)$ and $t_{ij}^{*}$ is the failure time of member *j* of cluster *i*. Then the observed censoring indicator $\delta_{ij}$ is equal to 1 if $c_{ij}>t_{ij}^{*}$, and 0 otherwise. Conditional on the frailty term $\nu_{i}$ and the covariate $x_{ij}$, the shared frailty model at time *t* of the jth observation of cluster *i* is defined in terms of a conditional hazard model [26]

(2) $$\lambda_{ij}\left(t|x_{ij},\nu_{i}\right)=\nu_{i}\lambda_{0}\left(t\right)exp\left\{x_{ij}^{\prime }\beta \right\},$$

where $\lambda_{0}\left(.\right)$ is the baseline hazard function, and β is a vector of the regression parameters. If $n_{i}=1$ for i=1,2,…,m, then the survival function in Expression (1) and the hazard function in Expression (2) reduce to a proportional hazard model [27]. The frailty effects $\nu_{i},i=1,\cdots ,m$ are assumed to be independent and identically distributed random variables with distribution functions such as log-normal, positive stable distribution, Gamma and inverse Gaussian distributions [25,26]. However, the Gamma and the inverse Gaussian distributions are the most common and widely used in the literature for determining the frailty effect, which acts multiplicatively on the baseline hazard. Due to their computational convenience, the Gamma and the inverse Gaussian distributions were used as the frailty distributions for this study.

#### 2.5.2. A Shared Gamma Frailty Model

The two parameter Gamma density function for the frailty term $\nu_{i}$ with shape parameters *k* and scale parameter λ are given by

$$f_{\nu }\left(\nu_{i}\right)=\frac{k^{\lambda }\nu_{i}^{\lambda -1}e^{-k\nu_{i}}}{\Gamma (\lambda )};\;\lambda >0,k>0.$$

The Laplace transformed version of this density has a form  [26]:

$$L\left(s\right)=\int_{0}^{\infty }exp\left(-\nu_{i}s\right)f_{\nu }\left(\nu_{i}\right)d\nu_{i}=k^{\lambda }{\left(s+k\right)}^{-\lambda }$$

The solutions of the first and second partial derivatives of the Laplace function L(.) with respect to *s* equal to 0 give the mean and variance of the frailty term $\nu_{i}$, $\mu_{\nu }=E\left(\nu_{i}\right)=\frac{k}{\lambda }$ and $\sigma_{\nu }^{2}=Var\left(\nu_{i}\right)=\frac{k}{\lambda^{2}}$, respectively. For identifiability reasons, we set the mean $\mu_{\nu }$ of the frailty term equal to 1 and the variance of the frailty term $\sigma_{\nu }^{2}$ becomes $\theta =\frac{1}{\lambda }$, which is similar to the one-parameter Gamma distribution. The shared Gamma frailty model (conditional hazard) for individual *j* in cluster *i* is then given by:

$$\lambda \left(t|x_{ij},\nu_{i}\right)=\nu_{i}\lambda_{0}\left(t\right)exp\left\{x_{ij}^{\prime }\beta \right\}=\nu_{i}\lambda \left(t_{ij}\right),$$

where, $\lambda \left(t_{ij}\right)=\lambda_{0}\left(t\right)exp\left\{x_{ij}^{\prime }\beta \right\}$ is the hazard function and $\nu_{i}$ is a Gamma distributed frailty variate. For the shared Gamma frailty model, Kendall’s τ is given by [26] (see Section 4.2)

$$\tau =\frac{\theta }{\theta +2},$$

where, $\tau \in \left(0,1\right)$ measures the correlation between any two event times within clusters.

#### 2.5.3. The Inverse Gaussian Frailty Model

The inverse Gaussian (inverse normal) distribution was introduced as a frailty distribution alternative to the Gamma distribution by Hosmer [28]. Similar to the Gamma frailty model, simple closed-form expressions exist for the unconditional survival and hazard functions, which makes the model attractive. The probability density function of an inverse Gaussian distributed frailty random variable $\nu_{i}$ with parameters μ>0 and α>0 is given by [26] (see Section 4.3),

$$f_{\nu }\left(\nu_{i}\right)=\frac{\alpha^{1/2}}{\sqrt{2\pi }}\nu_{i}^{-\frac{3}{2}}exp\left\{-\frac{\alpha }{2\nu_{i}\mu^{2}}{\left(\nu_{i}-\mu \right)}^{2}\right\},$$

where, the Laplace transform of this function has a form

$$L\left(s\right)=exp\left\{\frac{\alpha }{\mu }-{\left({\left(\frac{\alpha }{\mu }\right)}^{2}+2\alpha s\right)}^{1/2}\right\};s\geq 0.$$

Then, the mean is $\mu_{\nu }=-\frac{\partial L(s)}{\partial s}{|}_{s=0}=\mu$ and variance $\sigma_{\nu }^{2}=\frac{\partial^{2}L\left(s\right)}{\partial s^{2}}{|}_{s=0}-{\left(-\frac{\partial L(s)}{\partial s}{|}_{s=0}\right)}^{2}=\frac{\mu^{3}}{\alpha }.$ We set $\mu_{\nu }=1$ for identifiability reasons and $\theta =\sigma_{\nu }^{2}=\frac{1}{\alpha }.$ If there is no heterogeneity among clusters, then θ=0, which corresponds to α=+∞. For the inverse Gaussian frailty distribution, the conditional survival function is given by:

$$S_{\theta }\left(t\right)=exp\left\{\frac{1}{\theta }\left(1-{\left[1-2\theta ln\left(S\left(t\right)\right)\right]}^{1/2}\right)\right\},$$

and the conditional hazard function is given by:

$$h_{\theta }\left(t\right)=h\left(t\right){\left[1-2\theta ln(S(t))\right]}^{-\frac{1}{2}},$$

where, S(t) and h(t) represent the survival function and the hazard function of the baseline distributions, respectively. Kendall’s τ is then given by [29]:

$$\tau =\frac{1}{2}-\frac{1}{\theta }+2\frac{exp(2/\theta )}{\theta^{2}}\int_{2/\theta }^{\infty }\frac{exp(-\nu )}{\theta^{2}}d\nu ,$$

where, τ∈(0,1/2). In order to investigate the effect of the candidate covariates on the time to death among TB patients, univariable analysis was performed by fitting a separate model for each candidate covariate. Follow-up multivariable survival analysis was performed assuming the Weibull, log-logistic and the log-normal distributions for the baseline hazard function, and the Gamma and inverse Gaussian distributions for the frailty variate.

### 2.6. Parameter Estimation

For the right-censored clustered survival data, the observation for subject *j*, $j\;=\;1,\ldots ,n_{i}$ from cluster *i*, i=1,…,m is the couple $\left(t_{ij},\delta_{ij}\right)$, where $t_{ij}=min\left(t_{ij}^{*},c_{ij}\right)$ is the minimum between the survival time $t_{ij}^{*}$ and the censoring time $c_{ij}$. The event indicator variable $\delta_{ij}$ is defined as 1 for $t_{ij}^{*}\leq c_{ij}$, otherwise, $\delta_{ij}=0$, indicating right-censored observation. Since, the couple $\left(t_{ij},\delta_{ij}\right)$ constitute clustered survival data, frailty models account for the clustering present in grouped survival time data [26]. Let $\Omega =(\beta ,\Lambda_{0})$ be the vector of parameters, where β is the coefficient of covariates, and $\Lambda_{0}$ is the baseline hazard function. Then, the marginal-likelihood function of the observed data $\left\{\left(t_{ij},\delta_{ij},x_{ij}\right),i\in m,j\in n_{i}\right\}$ can be obtained by integrating out the conditional likelihood function with respect to the frailty variate, which is given by

(3) $$L\left(\Omega \right)=\prod_{i=1}^{m}\int \prod_{j=1}^{n_{i}}{\left(\lambda_{ij}\left(t|x_{ij},\nu_{i}\right)\right)}^{\delta_{ij}}S_{ij}\left(t|x_{ij},\nu_{i}\right)f_{\nu }\left(\nu_{i}\right)d\nu_{i}$$

where, $\lambda_{ij}\left(.\right)$ and $S_{ij}\left(.\right)$ are specified as in Expression (1) and (2), respectively. The parameters of frailty models Ω in Expressions (1) and (2) with shared Gamma and inverse Gaussian frailty are then estimated by maximizing (3). Applying the Laplace transform property, let the mth derivative of the Laplace transform evaluated at $H_{i.}\left(t\right)$ be given by

$$L^{\left(N_{i.}\left(t\right)\right)}(H_{i.}\left(t\right)={\left(-1\right)}^{N_{i.}\left(t\right)}\int \nu^{N_{i.}\left(t\right)}exp\left\{-\nu H_{i.}\left(t\right)\right\}f\left(\nu \right)d\nu ,$$

then the marginal log-likelihood in Expression (3) can be expressed as [29]:

(4) $$\begin{array}{cc} l_{marg}\left(\Omega \right)=\sum_{i=1}^{m} & \{\sum_{j=1}^{n_{i}}\left[\delta_{ij}\left(log\left(\lambda_{0}\left(t_{ij}\right)\right)+x_{ij}^{{}^{\prime }}\beta \right)\right]+\\ & log\left({\left(-1\right)}^{d_{i.}\left(t\right)}L^{d_{i.}\left(t\right)}\left(H_{i.}\left(t\right)\right)\right)\}, \end{array}$$

where, $d_{ij}\left(t\right)=\delta_{ij}I\left(t_{ij}\leq t\right)$ is the event indicator for the jth observation in the ith cluster, $d_{i.}\left(t\right)=\sum_{j=1}^{n_{i}}d_{ij}\left(t\right)$ is the number of events in the ith cluster, $H_{ij}\left(t\right)=\Lambda_{0}\left(t_{ij}\right)exp\left(x_{ij}^{\prime }\beta \right)$, and $H_{i.}\left(t\right)=\sum_{j=1}^{n_{i}}H_{ij}\left(t\right)$. Then, parameter estimates of β, θ and $\Lambda_{0}$, are obtained, maximizing the marginal log-likelihood (4) by computing the derivatives of L(.) up to the order of $q=max\{d_{1},\ldots ,d_{m}\}$. Although there are various types of R-package available, the parfm package [29] was used for estimating the parameters. The estimates and standard errors of the parameters of interest can be obtained from the parfm package. The acceleration factor (ϕ=exp(β)) measures the risk effect of covariates on the event of interest and was computed manually for the significant covariates. The most important issue is the interpretation of the acceleration factor; if 1 is not included in the acceleration confidence interval then the factors are statistically significant or else non-significant.

### 2.7. Models Comparison and Selection

There are several methods of model selection. In this study, the AIC [30] criterion was used to compare various candidate models and the model with the smallest AIC value was considered as having a better fit [29].

## 3. Results

### 3.1. Patients’ Characteristics

In this study, the time to death of TB patients was collected from a total of 604 patients identified retrospectively who had undergone anti-TB treatment. Complete follow-up information was available for 558 (92.4%) TB patients with mean (SD) age at baseline 42.78(16.01) years and median age 43 years. It was observed that 46 (7.6%) TB patients died during the follow-up period and the rest of the TB patients were censored at the end of the study. Regarding the sex composition of patients, 300 (49.7%) were males, among them 19(6.3%) died, while 27 (8.9%) deaths occurred among the female patients (Table 1). It was also observed that 282 (46.7%) patients were HIV-positive, of whom 27 (9.6%) later died. In addition, of 232 (38.4%) pulmonary TB patients, 15 (6.5%) died during the study period, while 25 (7.2%) deaths occurred among the negative pulmonary TB patients. Furthermore, 339 (56.1%) TB patients had no previous treatment history at baseline, of whom 28 (8.3%) later died.

Table 2 presents descriptive summaries for the continuous covariates. The median follow-up time was four months for patients that were censored and about 25% of the patients had three months of follow-up (lower quartile). The median time of death was five months. This indicates that most of the events or deaths occurred in the earlier months of the anti-TB treatment. At the start of the study, the baseline average (SD) age and weight of TB patients was 42.78(18.64) and 40.78(16.01), respectively. Fifty percent (50%) of the sampled patient ages fell between a lower quartile of 27.75 and an upper quartile of 58.00, whereas the patients’ weight fell between a lower quartile of 32.00 and an upper quartile of 53.00.

### 3.2. Comparison of Survival Curves

**Figure 1:**
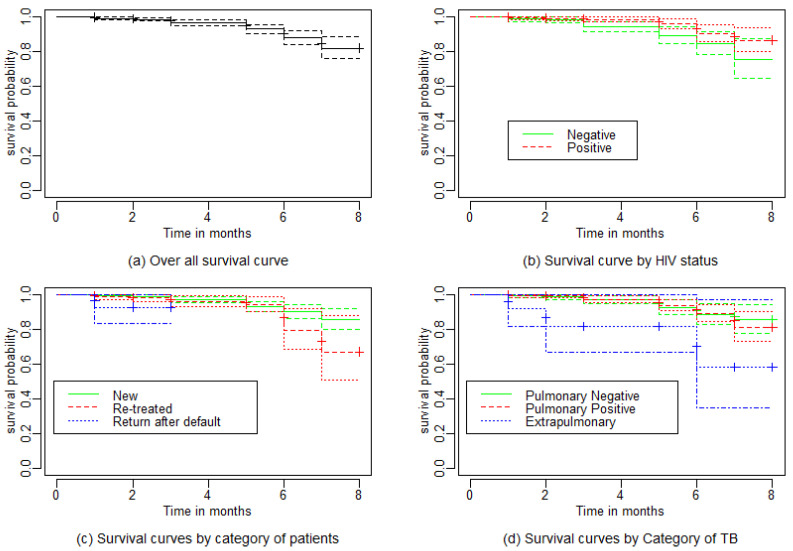
The Kaplan–Meier survival function plots for time to death of TB patients by (**a**) overall (**b**) HIV status (**c**) type of TB (**d**) category of TB.

The survival distributions of time to death of the TB patients were estimated for each group using the Kaplan–Meier (KM) method to compare the survival curves of two or more groups. The Kaplan–Meier estimated survival curves in Figure 1 highlight the overall estimated survival function using different groups of covariates. The overall estimated survival curve for the time to death of TB patients is shown in Figure 1a. In addition, the survival curves of TB patients were different for HIV status, smear result, category of TB, and type or status of TB patient (see Figure 1b–d), whereas among gender, previous treatment history, and place of residence there were not clear differences (figure not shown).

The observed differences in survival experiences in different patient groups was also assessed using the log-rank and Breslow tests [23]. Table 3 shows that there was a significant survival time difference among type of TB, patient category, and HIV status for TB patients at the 5% significance level. Since the null hypothesis was rejected for the covariates, type of TB and category of TB, post hoc analysis was performed using pairwise comparisons among the categories. The survival times or time to death of TB patients was not significantly different between pulmonary negative and pulmonary positive TB patients (*p*-value =0.90308), whereas extrapulmonary patients experienced significantly different survival times when compared to pulmonary negative and pulmonary positive patients (*p*-value =0.00077). The interactive effects of gender, smear result, previous treatment history, and place of residence with survival time of TB patients were statistically non-significant (result not shown).

### 3.3. Model Selection and Inference

The Weibull, log-logistic, and log-normal distributions for baseline hazard function were also assessed. The likelihood ratio test (LRT) result for testing the regression parameters equal to zero was rejected for all baseline hazard distributions. The LRT, parameter estimates, standard error, and *p*-value of the models are displayed in Table 4. The parameter estimates obtained were consistent in sign for all the baseline distributions. However, the standard error was relatively smaller for the Weibull baseline distribution when compared to the other baseline distributions. The age, weight, HIV status (positive), smear result (positive), extrapulmonary TB type, retreated, and return-after-default were important covariates related to the survival of TB patients. The loglikelihood value for the loglogistic baseline was −195.665 with an LRT chi-square value of $\chi^{2}=38.3$ and a *p*-value <0.0001. For the Weibull and lognormal baseline, the loglikelihood values were −194.797 and −199.025, respectively. It was observed that the Weibull baseline had the highest loglikelihood value of −194.797, indicating that the Weibull baseline was better fitted to the data than the others. The survival model fitted with the Weibull baseline was significant with $\chi^{2}=39.2$ and *p*-value <0.0001.

The Gamma and inverse-Gaussian distributions are the most common and widely used distributions cited in the literature for modeling the frailty effect [25,26]. Thus, considering the Gamma and inverse-Gaussian distributions for frailty random variates (hospitals), parametric shared frailty models were fitted. The parameter estimates, standard error (se) and *p*-value (*p*) of Gamma and inverse-Gaussian shared frailty models are displayed in Table 5. The loglikelihood value for the Gamma frailty model with Weibull distribution for baseline hazard function was −192.17, which was the largest among the models considered. This indicated that the Weibull baseline hazard distribution for both frailty variates fitted better for the time to death outcomes of TB patients. The Weibull–Gamma shared frailty model had a minimum AIC value of 406.34 compared to the other models, suggesting that it is the preferred model for describing the TB dataset (see, Table 5).

The heterogeneity test of the frailty terms for the selected model was performed using the one-sided likelihood ratio test (LRT) proposed by Claeskens et al. [31]. The LRT for testing $H_{o}:\sigma_{\tau }^{2}=0$versus$H_{a}:\sigma_{\tau }^{2}>0$ for a shared Gamma frailty model with a Weibull baseline hazard function has an asymptotic $\frac{1}{2}\chi_{0}^{2}+\frac{1}{2}\chi_{1}^{2}$ mixture distribution. The loglikelihood value for the null model (Weibull baseline model) was −194.797, and for the full model (Gamma frailty model) was −192.17, with an observed value for the LRT statistic of 5.254 with a *p*-value of 0.012. Thus, the variances of the random effect of the Gamma shared frailty model were significant at the 5% level of significance.

This result indicates the existence of unobserved heterogeneity between the hospitals and that the frailty component in the model was important. The heterogeneity estimate was θ=4.237 and the dependence within the clusters (treatment centres) was measured by Kendall’s tau as $\hat{\tau }=0.106$ (see, Table 5). Therefore, the time to death of TB patients was modeled by a Gamma shared frailty model with hospitals as a clustering effect and a parametric Weibull distribution for the baseline hazard function.

The parameter estimate, standard error, and *p*-value for the Weibull–Gamma shared frailty model displayed in Table 5 show that age in years, initial weight, category of TB patient, extrapulmonary type of TB, and HIV status were important factors affecting the time to death of TB patients. However, the variables, gender, smear result, previous treatment history, and place of residence had no significant effect on the time to death of TB patients.

Conditional on the hospital frailty, the estimated acceleration factor or risk of death for the age of TB patients was exp(0.022)≈1.022, with a 95% confidence interval (CI) of (1.004,1.040). After adjusting for other covariates, a one year increase in the age of a TB patient increased the risk of death by 1.022 times, whereas, an increase in age of 10 years resulted in an increased risk of death by exp(0.22)≈1.2461 times. These results imply that the risk of death was higher for older TB patients compared to younger patients.

The estimated acceleration factor and its 95%CI for initial weight were exp(−0.038)≈0.9627 and (0.9400, 0.9805), respectively. For a one kg increase in the weight of a TB patient, the risk of death decreased by 0.9627 times, holding the effects of other covariates constant and accounting for frailty effects. Thus, patients with low weight measurements at baseline had a higher risk of death.

The hazard rate or risk of death for extrapulmonary TB patients was significantly different from that for pulmonary negative TB patients (*p*-value =0.006), while the risk of death was not significantly different for pulmonary positive patients. The estimated acceleration factor for extrapulmonary TB patients at any time *t* was exp(1.456)≈4.289, with 95% Wald CI (1.534, 11.993). Thus, conditional on the frailty effects, the risk of death for extrapulmonary TB patients was increased by 4.289 times compared to that of pulmonary negative patients, holding the effects of other covariates constant.

The estimated acceleration factor and its 95% CI for HIV-negative TB patients were exp(−0.801)≈0.4489 and (0.247,0.817), respectively. The point estimate indicates that HIV-negative TB patients were about 0.4489 times less likely to die than HIV-positive TB patients. This indicates that HIV-negative patients had a higher survival probability compared to HIV-positive patients.

According to the result of the Weibull–Gamma shared frailty model, the estimated acceleration factor for re-treated TB patients was exp(0.760)≈2.1383 with a 95% CI of (1.130,4.061), while, for returned TB patient’s after treatment, the default was exp(2.588)≈13.301 with a 95% CI of (2.729,65.281). Thus, conditional on the covariate and frailty effect, the hazard or risk of death for retreated TB patients at any time *t* was 2.1383 times that of newly diagnosed TB patients, while TB patients who returned after treatment default were 13.301 times more likely to die compared to new TB patients.

## 4. Discussion

Analysis of survival data in which the response variable is time to death has many applications in public health. In most public health studies, survival data are collected from different groups (clusters). In most cases, subjects in the same group with similar risk factors behave as if they were subjects in different groups. In such situations, convectional survival models do not capture the complete dependency between the observations in the model. Another approach is to use a parametric shared frailty model. This model is a random effects model where the frailties are common among groups of different individuals and are randomly dispersed across groups [22]. In this study, shared frailty models were used, assuming that patients in the same hospital may be more similar than others due to common risk factors for shared illnesses, such as air pollution, medical facilities, access to medical care, and altitude. Therefore, the current study applied a parametric shared frailty model to estimate the time to death of tuberculosis patients.

The study findings indicated that the treatment success rate of TB patients treated under the DOTs program was 92.4% (14.6% cure rate and 77.4% treatment completion rate). These figures are higher than those presented in the 2020 WHO global tuberculosis report on Africa [3], and a study undertaken in Tigray [32], which reported a treatment success rate of 89%. However, the current finding is consistent with [33] that reported a success rate of 91.7%. The results indicate that the median follow-up time was six months for censored patients where 25% of the patients had four months of follow-up (lower quartile). Further, the median time of death was four months which was greater than the result obtained by [32] in northern Ethiopia, in the Tigray region. This indicates that most of the events or deaths occurred in the earlier months after anti-TB treatment was initiated. This could have been due to the delayed presentation and diagnosis of TB cases resulting in a spreading out of the disease and reported early TB death.

The Kaplan–Meier survival curves show that the time to death of TB patients was different among HIV status, smear result, type of TB, and type of patient categories. However, clear differences were not observed among covariate gender, previous treatment history, and place of residence of TB patients. This result is consistent with that of [11]. The log-rank and Breslow tests also showed that there was a significant difference among the category of patients. Similarly, the Kaplan–Meier survivor estimates for type of TB, HIV status, and type of patient categories showed significance differences. These findings are consistent with those of a study performed in Addis Ababa in selected health centers [17], where it was found that the survival experience of patients in different categories of tuberculosis, and of HIV status differed significantly.

The multivariable analysis undertaken indicated that the covariates age, initial weight, type of patient, HIV status, smear result, and extrapulmonary type of TB were significantly associated with the time to death of TB patients. However, gender, place of residence, pulmonary positive type of TB patient, and previous treatment were not significantly associated with the time to death of patients. These results are consistent with the findings of [19,34]. According to the result obtained from the multivariable Weibull–Gamma shared frailty model, the risk of death was higher in HIV-positive co-infected TB patients and was statistically associated with the time to death of TB patients during treatment. This finding is consistent with those of a study conducted in the southern region of Ethiopia [35].

The findings from the current study suggest that the risk of death of TB patients increased with age. Thus, a one year increase in the age of TB patients increased the risk of death by 2.2%. This result implies that the age of patients was directly associated with the mortality of TB patients [35,36,37]. Similarly, [38] showed that mortality due to TB was indirectly associated with increasing age. With respect to the weight of patients, a one kg increase in the weight of a TB patient was found to decrease the risk of death by 3.7%. Thus, patients with low weight measurements at baseline had a higher risk of death. The result concerning the initial weight of TB patients from the current study is consistent with that obtained in a study conducted in the Dangila Woreda in northwest Ethiopia [39]. Similarly, a study in Singapore [8] showed that patients weighing less than 35 kg at the initiation of TB treatment were 3.7 times more likely to die during TB treatment [40].

The risk of death for extrapulmonary TB patients was significantly different from that of pulmonary negative TB patients. This result is consistent with that of [41]. Further, the covariate, gender, was found not to be a statistically significant factor affecting the survival of TB patients. However, other studies have reported that male TB patients were at higher risk of dying due to TB [18,19,40], and that type of tuberculosis (positive pulmonary, negative pulmonary, and extrapulmonary tuberculosis) were significant predictive factors of mortality of tuberculosis patients [17,20,40].

The results of this study show that HIV-positive TB patients were more likely to die when compared to HIV-negative TB patients. This may indicate that HIV-positive patients had lower survival probability than HIV-negative TB patients. This result is consistent with [42,43], but contradicts the findings of [44]. This might be due to differences in the socio-demographic characteristics of the patients.

## 5. Limitation and Importance of the Study

Since a retrospective cohort design was applied, the study was limited to data extracted from individual patient admission registry and medical logbooks. Hence, the study was subject to selection bias. There are various risk factors for the causes of death of tuberculosis patients during the DOTs program. In this study, the statistical analyses included all types of death observed, irrespective of the cause of death; therefore, there might be misclassification of the causes of death. In addition, there was incomplete information for some important demographic, as well as clinical, variables; thus, the study was limited to only those variables with complete information described in the Methods section. Despite these limitations, this study is very important for the follow up of TB patients by physicians during the intensive phase and of patients from higher age groups, and may contribute to reducing deaths during TB treatment. Overall, the results should serve to encourage other health professionals to undertake related studies in other parts of the country, and should also encourage programme staff to carefully record clinical variables with complete information.

## 6. Conclusions

The survival probability of older adult TB patients was lower compared to younger patients. Most TB deaths occurred in the first four months of anti-TB treatment. There was a substantially higher survival probability in young patients, those with normal baseline body weight, and for non-HIV-co-infected patients. Further, HIV-positive patients had a higher risk of early death during treatment than HIV-negative patients. Therefore, health care professionals should strengthen the DOTs program and consider the status of patients to maximize health benefits.

## Figures and Tables

**Table 1:** Characteristics of tuberculosis patients under DOTS program from randomly selected governmental hospitals in southwest Ethiopia.

		Censored	Death	Total		
Covariates	Categories	N (%)	N (%)	N (%)	Mean	Median
Sex	Male	281(93.7)	19(6.3)	300(49.7)	4.286	3
	Female	277(91.1)	27(8.9)	304(50.3)	4.098	3
Place	Rural	517(92.3)	43(7.7)	560(92.7)	4.196	3
	Urban	41(93.2)	3(6.8)	44(7.3)	4.136	3
HIV status	Negative	303(94.1)	19(5.9)	322(53.3)	3.865	5
	Positive	255(90.4)	27(9.6)	282(46.7)	4.478	3
Smear results	Negative	334(90.3)	36(9.7)	370(61.3)	4.254	5
	Positive	224(95.7)	10(4.3)	234(38.7)	4.094	3
Type of TB	Pulmonary positives	217(93.5)	15(6.5)	232(38.4)	4.296	3
	Pulmonary negative	323(92.8)	25(7.2)	348(57.6)	4.116	5
	Extrapulmonary	18(75.0)	6(25.0)	24(4.0)	3.416	5
Categories of TB	New	354(92.7)	28(7.3)	382(63.2)	4.662	5
	Retreated	179(91.8)	16(8.2)	195(32.3)	3.589	3
	Return after default	25(92.6)	2(7.4)	27(4.5)	1.888	2
History of previous	Yes	311(91.7)	28(8.3)	339(56.1)	4.395	5
treatment	No	247(93.2)	18(6.8)	265(43.9)	3.932	3

**Table 2:** Summary statistics of follow-up time in months and status of TB patients in randomly selected governmental hospitals in southwest Ethiopia.

Outcome		N	Percent	Mean	SD	Median	IQR
Status of patients							
	Censored	558	92.4	4.19	2.00	4.00	[3.00, 6.00]
	Dead	46	7.60	4.26	2.00	5.00	[3.00, 6.00]
Baseline age		604		42.78	18.64	43.00	[27.75, 58.00]
Baseline weight		604		40.78	16.01	35.00	[32.00, 53.00]

**Table 3:** Chi-square estimate ($\chi^{2}$) and *p*-value of log-rank and Breslow tests for comparing the survival experience of TB patients from randomly selected governmental hospitals in southwest Ethiopia.

		Log Rank Test	Breslow Test
Covariates	Categories	$\chi^{2}$ (*p*-Value)	$\chi^{2}$ (*p*-Value)
Sex	Male	2.22 (0.135)	2.21 ( 0.139)
	Female		
Place	Rural	0.03 ( 0.857)	0.03 (0.857)
	Urban		
HIV status	Negative	6.20 (0.012)	6.36 (0.011)
	Positive		
Smear results	Negative	5.31 (0.021)	4.90 (0.030)
	Positive		
Type of TB	Pulmonary negative	14.14 (0.009)	22.10 (0.000)
	Pulmonary positives		
	Extra pulmonary		
Category of TB	New	7.47 (0.023)	6.26 ( 0.045)
	Retreated		
	Return after default		
History of previous	Yes	0.001 (0.979)	0.34 (0.559)
treatment	No		

**Table 4:** Parameter estimates, standard error (se) and *p*-value (*p*) of survival models with parametric baseline hazard distribution for time to death of TB Patients.

	Weibull	Loglogistics	lognormal
Covariates	Estimate (se, *p*)	Estimate (se, *p*)	Estimate (se, *p*)
Sex (Male)	−0.249 (0.311, 0.423)	−0.221 (0.314, 0.481)	−0.198 (0.316, 0.531)
Age in years	0.015 (0.008, 0.054)	0.017 (0.008, 0.035)	0.017 (0.008, 0.029)
Weight in Kg	−0.030 (0.011, 0.004)	−0.025 (0.011, 0.019)	−0.022 (0.011, 0.040)
Residence (Rural)	0.088 (0.535, 0.869)	0.348 (0.628, 0.579)	0.507 (0.698, 0.468)
HIV status (Negative)	−0.840 (0.303, 0.006)	−0.815 (0.305, 0.007)	−0.783 (0.306, 0.010)
Smear results (Positive)	−0.892 (0.365, 0.015)	−0.858 (0.367, 0.019)	−0.843 (0.368, 0.022)
Type of TB			
Pulmonary positive	0.037 (0.322, 0.909)	0.088 (0.329, 0.790)	0.114 (0.333, 0.733)
Extrapulmonary	1.642 (0.508, 0.001)	1.661 (0.511, 0.001)	1.699 (0.514, 0.001)
Category of patient			
Re-treated	0.653 (0.322, 0.042)	0.653 (0.322, 0.043)	0.618 (0.322, 0.055)
Return after default	2.466 (0.795, 0.002)	2.469 (0.805, 0.002)	2.323 (0.794, 0.003)
Previous treatment history (Yes)	−0.233 (0.327, 0.477)	−0.161 (0.334, 0.630)	−0.116 (0.336, 0.729)
Loglikelihood	−194.797	−195.665	−199.025
Likelihood ratio test	($\chi^{2}$ = 39.2 p< 0.0001)	($\chi^{2}$ = 38.3, p< 0.0001)	($\chi^{2}$ = 36.9, p< 0.0001)

**Table 5:** Parameter estimates, standard error (se) and *p*-value (*p*) of Gamma and inverse-Gaussian shared frailty models for time to death of TB Patients.

	Weibull	Loglogistics	Lognormal
Covariates	Estimate (se, *p*)	Estimate (se, *p*)	Estimate (se, *p*)
Gamma frailty model			
Sex (Male)	−0.458 (0.328, 0.163)	−0.407 (0.333, 0.221)	−0.376 (0.334, 0.261)
Age in years	0.022 (0.009, 0.014)	0.023 (0.009, 0.008)	0.023 (0.009, 0.009)
Weight in Kg	−0.038 (0.012, 0.001)	−0.032 (0.011, 0.004)	−0.028 (0.011, 0.012)
Residence (Rural)	0.030 (0.564, 0.958)	0.374 (0.640, 0.558)	0.520 (0.713, 0.465)
HIV status (Negative)	−0.801 (0.305, 0.009)	−0.777 (0.307, 0.011)	−0.744 (0.307, 0.015)
Smear results (Positive)	−0.634 (0.386, 0.101)	−0.611 (0.386, 0.114)	−0.615 (0.387, 0.112)
Type of TB			
Pulmonary positive	0.049 (0.327, 0.880)	0.104 (0.332, 0.755)	0.131 (0.335, 0.696)
Extrapulmonary	1.456 ( 0.525, 0.006)	1.505 (0.525, 0.004)	1.567 (0.525, 0.003)
Category of patient			
Re-treated	0.762 (0.326, 0.020)	0.758 (0.328, 0.021)	706 (0.327, 0.031)
Return after default	2.591 (0.810, 0.001)	2.593 (0.818, 0.002)	2.437 (0.805, 0.002)
Previous treatment history (Yes)	−0.226 (0.331, 0.494)	−0.144 (0.332, 0.664)	−0.100 (0.334, 0.765)
Kendal’s τ	0.106	0.098	0.09
Loglikelihood	−192.17	−193.386	−197.062
Likelihood Ratio Test	($\chi^{2}$ = 5.254, p< 0.012)	($\chi^{2}$ = 4.123, p< 0.000)	($\chi^{2}$ = 3.421, p< 0.000)
Inverse-Gaussian frailty model			
Sex (Male)	−0.454 (0.329, 0..168)	−0.405 (0.334, 0.225)	−0.373 (0.335, 0.266)
Age in years	0.021 (0.009, 0.015)	0.023 (0.009, 0.009)	0.023 (0.009, 0.009)
Weight in Kg	−0.038 (0.012, 0.001)	−0.032 (0.011, 0.005)	−0.028 (0.011, 0.012)
Residence (Rural)	0.031 (0.568, 0.956)	0.372 (0.639, 0.560)	0.519 (0.713, 0.466)
HIV status (Negative)	−0.802 (0.305, 0.009)	−0.778 (0.307, 0.011)	−0.745 (0.307, 0.015)
Smear results (Positive)	−0.640 (0.386, 0.097)	−0.616 (0.386, 0.111)	−0.619 (0.38, 0.110)
Type of TB			
Pulmonary positive	0.049 (0.327, 0.880)	0.104 (0.332, 0.755)	0.131 (0.335, 0.697)
Extrapulmonary	1.461 (0.526, 0.006)	1.509 (0.527, 0.004)	1.570 (0.527, 0.003)
Category of patient			
Re-treated	0.760 (0.327, 0.020)	0.756 (0.328, 0.021)	0.704 (0.327, 0.031)
Return after default	2.588 (0.811, 0.001)	2.591 (0.818, 0.002)	2.435 (0.805, 0.002)
Previous treatment history (Yes)	−0.227 (0.331, 0.494)	−0.145 (0.332, 0.662)	−0.101 (0.334, 0.763)
Kendal’s τ	0.104	0.092	0.084
Loglikelihood	−192.219	−193.421	−197.089
Likelihood Ratio Test	($\chi^{2}$ = 8.143 p< 0.000)	($\chi^{2}$ = 6.213, p< 0.000)	($\chi^{2}$ = 3.456, p< 0.000)

## Data Availability

The datasets analyzed in this study are available from the corresponding author on reasonable request.

## Abbreviations

The following abbreviations are used in this manuscript:

AIC	Akaki Information Criteria
ART	Antiretroviral therapy
CI	Confidence interval
DOTS	Directly observed treatment, short-course
EPTB	Extrapulmonary tuberculosis
HIV	Human immunodeficiency virus
MOH	Ministry of Health
TB	Tuberculosis
WHO	World Health Organization
